# A novel *NHS* mutation causes Nance-Horan Syndrome in a Chinese family

**DOI:** 10.1186/s12881-016-0360-9

**Published:** 2017-01-07

**Authors:** Qi Tian, Yunping Li, Rizwana Kousar, Hui Guo, Fenglan Peng, Yu Zheng, Xiaohua Yang, Zhigao Long, Runyi Tian, Kun Xia, Haiying Lin, Qian Pan

**Affiliations:** 1 State Key Laboratory of Medical Genetics & School of Life Sciences, Central South University, Changsha, Hunan China; 2 Department of Ophthalmology, The Second Xiangya Hospital, Central South University, Changsha, Hunan China; 3 Department of Biology, Allama Iqbal Open University, Islamabad, Pakistan; 4 ChangSha Health Vocational Collage, Changsha, Hunan China; 5 Shenzhen Baoan District Maternal and Child Health Hospital, Shenzhen, Guangdong China

**Keywords:** Congenital cataract, Exome sequencing, Sanger sequencing, Splice site, RT-PCR

## Abstract

**Background:**

Nance-Horan Syndrome (NHS) (OMIM: 302350) is a rare X-linked developmental disorder characterized by bilateral congenital cataracts, with occasional dental anomalies, characteristic dysmorphic features, brachymetacarpia and mental retardation. Carrier females exhibit similar manifestations that are less severe than in affected males.

**Methods:**

Here, we report a four-generation Chinese family with multiple affected individuals presenting Nance-Horan Syndrome. Whole-exome sequencing combined with RT-PCR and Sanger sequencing was used to search for a genetic cause underlying the disease phenotype.

**Results:**

Whole-exome sequencing identified in all affected individuals of the family a novel donor splicing site mutation (NM_198270: c.1045 + 2T > A) in intron 4 of the gene *NHS*, which maps to chromosome Xp22.13. The identified mutation results in an RNA processing defect causing a 416-nucleotide addition to exon 4 of the mRNA transcript, likely producing a truncated NHS protein.

**Conclusions:**

The donor splicing site mutation NM_198270: c.1045 + 2T > A of the *NHS* gene is the causative mutation in this Nance-Horan Syndrome family. This research broadens the spectrum of *NHS* gene mutations, contributing to our understanding of the molecular genetics of NHS.

**Electronic supplementary material:**

The online version of this article (doi:10.1186/s12881-016-0360-9) contains supplementary material, which is available to authorized users.

## Background

Nance-Horan Syndrome (NHS) (OMIM: 302350) was first described in detail by two independent studies in 1974 as a rare X-linked developmental disorder characterized by congenital cataracts, with occasional dental anomalies, brachymetacarpia and mental retardation [1–4]. It is inherited as an X-linked recessive trait, whereby heterozygous females manifest a similar but milder disease phenotype than affected males [5].

The *NHS* (Nance-Horan syndrome) gene has been mapped to chromosome Xp22.13 [6]. The gene is composed of 10 coding exons in an approximately 650-kb [7] region, encoding at least four isoforms as a result of alternative splicing. The two major isoforms, NHS-A and NHS-1A, are transcribed from exon 1 and comprise 1630 amino acids and 1651 amino acids, respectively. NHS-B, a 1335-amino acid protein, is transcribed from exon 1b; NHS-C, a 1453-amino acid protein, is transcribed from exon 1a [8–10].

To date, at least 33 mutations [11–15], including 6 from the Chinese population, have been reported in the *NHS* gene, many of which are nonsense mutations or InDels, with few copy number variations and missense and splice site mutations. In this study, we report the identification of a novel splice site mutation in the *NHS* gene in a Chinese family with NHS. This report broadens the spectrum of *NHS* gene mutations implicated in NHS pathogenesis.

## Methods

### Study subjects and clinical characterization

**Fig. 1:**
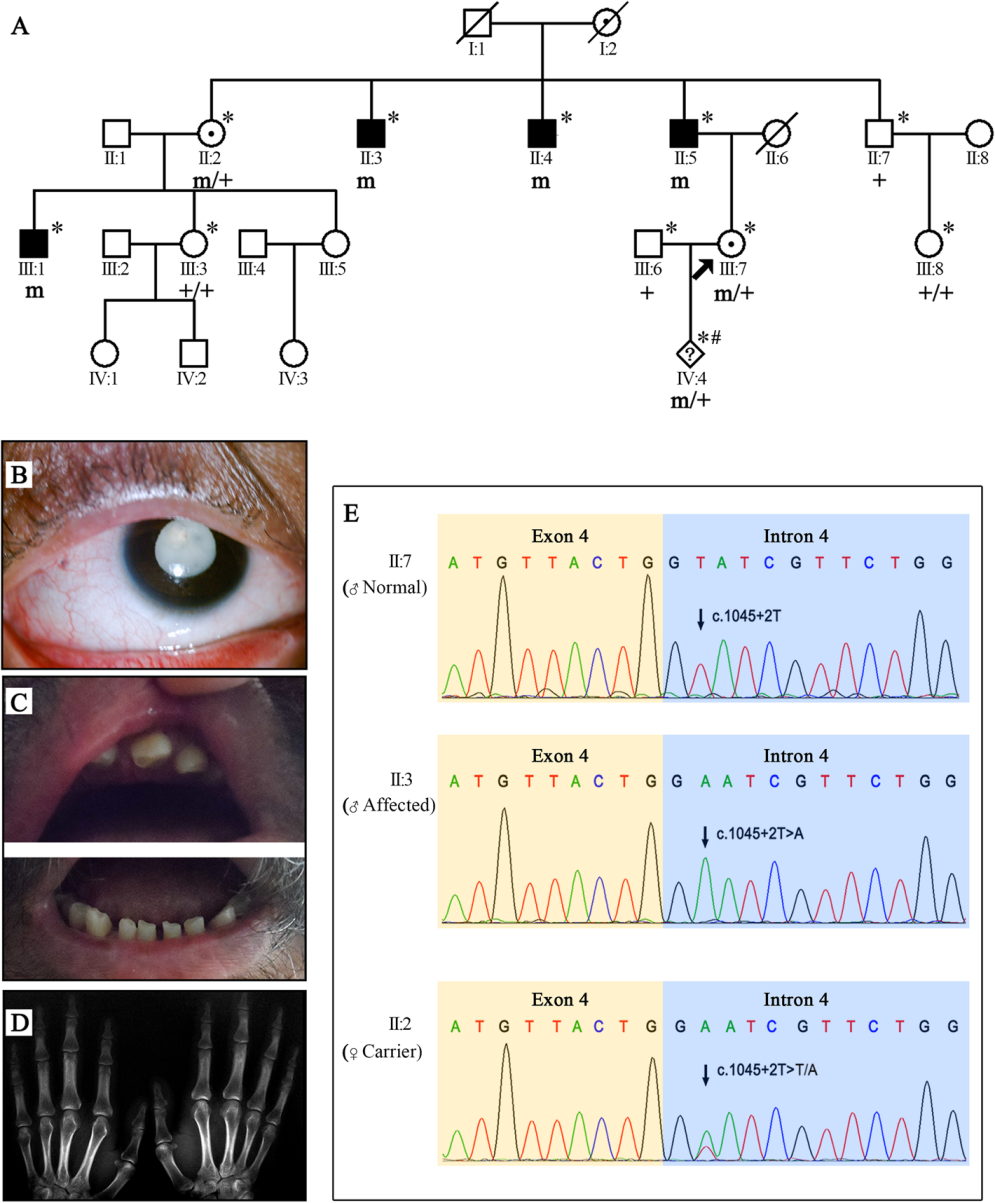
**a** The pedigree of the family with *NHS* gene mutations: clear and filled symbols represent unaffected and affected individuals, respectively. A dotted circle indicates an obligate X-linked carrier, whereas a “#” symbol indicates a fetus. Deceased individuals are indicated with a slash. Individuals with “*” were recruited and analyzed. “m” and “+” refer to mutant and normal alleles, respectively. **b** Photograph of the right eye of an affected male (III: 1), indicating a nuclear cataract. **c** Photograph of the teeth of an affected male (III: 1) presenting diastema (**d**). Radiographs of the right and left hands of an affected male (II: 5), indicating brachymetacarpia. **e** Sequence chromatogram of the Chinese family with the novel *NHS* gene mutation; the upper panel represents the nucleotide sequence of an unaffected male, the middle panel that of an affected male, and the lower panel that of a carrier female; an arrow indicates the site of mutation

A four-generation family with multiple affected individuals was recruited from Guangdong Province (Fig. 1a). Pedigree analysis suggests X-linked inheritance. Approval of this study was obtained from the Institutional Review Board (IRB) of the State Key Laboratory of Medical Genetics (SKLMG). All of the procedures were performed in accordance with the relevant policies in China and adhered to the tenets of the Declaration of Helsinki. After obtaining written informed consent, the affected members of this family were given a clinical examination at the Second Xiangya Hospital, Central South University, P.R.C. Medical and ophthalmic histories were recorded. Genomic DNA was extracted from venous blood of 6 affected and 4 normal family members; amniotic fluid was used for a fetus of uncertain status. Lymphoblastoid cell lines were established through Epstein-Barr virus (EBV) transformation of B-lymphocytes from an affected subject (II: 5), a carrier (II: 2) and a control.

### Whole-exome sequencing

We performed whole-exome sequencing of an affected male (II: 3) from this family. All procedures including read mapping and variant analysis were as described previously [16]. Variants predicted to alter the protein sequence were filtered as follows: 1) extraction of exonic non-synonymous single-nucleotide variants (SNVs), splice site SNVs and short insertions and deletions (InDel); 2) exclusion of high-frequency (MAF > 0.01) polymorphisms in the ESP6500 and 1000 Genomes databases; 3) extraction of variants in cataract-causing genes. Whole-exome sequencing was followed by Sanger sequencing to verify the variants detected.

### Sanger sequencing and RT-PCR

To validate the variants identified by the filtering procedure, Sanger sequencing using an ABI PRISM 3730 DNA Sequencer (Applied Biosystems, Foster City, CA) was performed by amplifying targeted genomic regions in candidate genes. Primers flanking the candidate variant in the *NHS* (NM_198270) and *PAX6* (NM_000280) genes were designed using Primer3 software (http://frodo.wi.mit.edu/). The *NHS* gene forward and reverse primers were 5′-TTTGTTTGCAGTCCCATCCC-3′ and 5′-GCCCGCGGAAAACATGTAAA-3′, respectively. The *PAX6* gene forward and reverse primers were 5′-TTCCACGGTGTATCTGCAAA-3′ and 5′-CAATGTGGTCGATGTGTCCC-3′, respectively. Co-segregation analysis between candidate genes and the disease phenotype was carried out using Sanger-sequenced DNA samples for all available family members. For reverse transcription-PCR (RT-PCR), RNA was extracted from lymphoblastoid cell lines established for two patients (II; 2, II; 5) and one control using the Trizol reagent (Thermo Fisher Scientific, 168 Third Avenue, Waltham, MA USA 02451), as described by the manufacturer. First-strand cDNA was synthesized using RevertAid™ First Strand cDNA Synthesis Kit (Thermo Fisher Scientific, 168 Third Avenue, Waltham, MA USA 02451) following the manufacturer’s instructions. RT-PCR for *NHS* cDNA was performed using primers for exon 4 (forward primer 5′-ACAGATGTCATGTTAGGGCAG-3′) and exon 6 (reverse primer 5′-TATCCTCGGTTTGGCACTCA-3′). Amplicons were visualized on 1.2% agarose gels stained with ethidium bromide. Sanger sequencing was performed to validate variations in mRNA sequences. Sequence analysis was performed by comparing sequence data with the reference genome using DNASTAR software.

## Results

### Clinical findings

Two affected male subjects (II: 5, III: 1) available at the time of clinical examination presented with bilateral congenital nuclear cataracts, one of whom received cataract surgery at the age of 30 years. Additional ocular features recorded in the affected subjects include microcornea, high myopia, nystagmus and strabismus. Brachymetacarpia was noted in both affected male subjects, and characteristic facial features of a long narrow face and prominent nose were exhibited by one male subject (II: 5). Mild mental retardation and diastema were observed in the other male subject (III: 1); the teeth of the other affected male (II: 5) could not be evaluated because of tooth loss due to aging. Heterozygous obligate carrier females (II: 2, III: 7) received cataract surgery a long time before the family was recruited for the present study. Nonocular features observed included brachymetacarpia in both carriers (II: 2, III: 7) and diastema in one (III: 7). A detailed description of the clinical features of the affected and carrier family members are summarized in Table 1.

**Table 1:** Clinical features of affected and carrier members of the NHS family

Disease phenotype		II:5 (affected)	III:1 (affected)	II:2 (carrier)	III:7 (carrier)
Ocular features	Bilateral congenital cataract	+	+	+	+
	Microcornea	+	+	+	+
	Nystagmus	+	+	+	+
	Microphthalmia	-	-	-	-
	High myopia	+	+	+	+
	Strabismus	+	+	+	+
Non-ocular features	Brachymetacarpia	+	+	+	+
	Prominent nose	+	-	-	-
	Long and narrow face	+	-	-	-
	Diastema	NA	+	NA	+
	Mental retardation	-	+	-	-

*NA* not available

### Exome sequencing identified an NHS splice site variation

Exome sequencing generated 4.93 Gb of sequence data, allowing the targeted region of the genome to be examined more than 50 times. A summary of the whole-exome sequencing results is provided in Additional file 1. Exome sequencing initially identified 24,403 coding SNVs and InDels. After the filtering procedure, 11,992 variants were retained, including exonic nonsynonymous and splice site mutations and InDels. Among these, 2226 variants were found to be low-frequency (MAF < 0.01) polymorphisms in the ESP6500 and 1000 Genomes databases (See Additional file 2). For this study, we focused only on variants detected in reported cataract-causing genes. These included c.G451A and c.1045 + 2T > A in the *PAX6* and *NHS* genes, respectively (See Additional file 3). These two variants were further validated by Sanger sequencing of 10 family members (including male member II: 3 who underwent exome sequencing) and one fetus. Only *NHS* gene mutation c.1045 + 2T > A was detected in all 6 patients but not in 4 unaffected family members and 200 control subjects. The fetus was identified as a heterozygote (m/+). The identified mutation causes a T-to-A transversion in the splice donor site of intron 4 of the *NHS* gene. The pathogenic sequence variant reported here was found in the heterozygous state (m/+) in obligate carrier females and in a hemizygous state (m) in affected males, co-segregating with the disease phenotype as an X-linked recessive trait.

### RT-PCR and sequencing

**Fig. 2:**
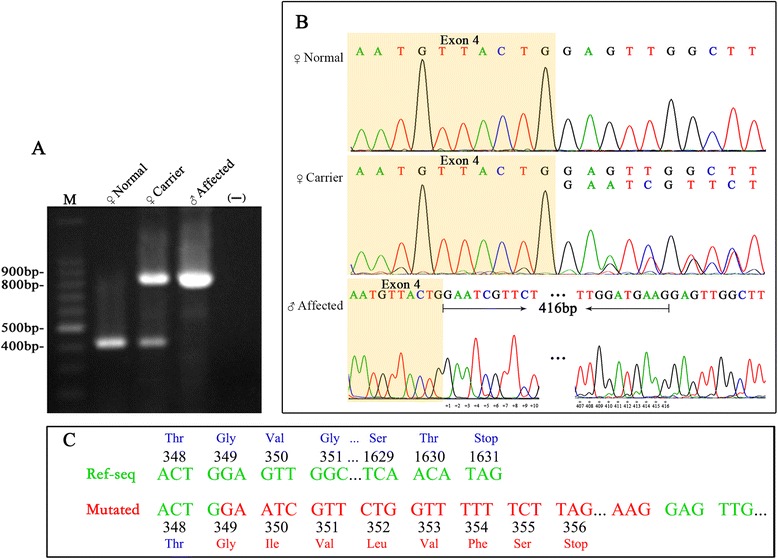
**a** RT-PCR analysis of the splice site mutation in the *NHS* gene. Samples were from a normal female, a carrier female and an affected male. The RT-PCR product separated by 1.2% agarose gel electrophoresis is a band of the expected size (421 bp) in the normal female and an aberrant band of 837 bp in the affected male (II: 5); bands of both sizes (421 bp and 837 bp) are observed in the carrier female (II: 2); “M” denotes the 100 bp ladder, and “–” represents the negative control. **b** Chromatogram of RT-PCR product sequencing. The upper panel represents the sequence for normal splicing in the control subject, the middle panel represents the sequence for normal and aberrant splicing in a carrier female (II: 2), the lower panel represents the sequence for aberrant splicing with insertion of 416 nucleotides in exon 4 in an affected male (II: 5). **c** Sequence of nucleotides and corresponding amino acids in the mutated NHS protein aligned with reference sequence; the premature stop codon is indicated

To assess whether the T > A substitution in the splice donor site of intron 4 of the *NHS* gene affects precursor mRNA splicing, we performed an RT-PCR experiment using RNA extracted from lymphoblast cell lines established for two patients (II: 2, II: 5) and one control. Agarose gel electrophoresis of the resulting PCR products showed amplicons of two different lengths. A shorter amplicon of approximately 421 bp detected in the control subject corresponded with the expected band size. An additional aberrant amplicon of 837 bp was detected in the carrier female (II: 2), whereas the affected male (II: 5) presented only an aberrant amplicon (837 bp) (Fig. 2a). Sequencing of the RT-PCR product revealed an insertion of 416 nucleotides (c.1045G_1046Gins416bp) in exon 4 due to abnormal splicing occurring at a cryptic splice site in the patients compared to the control (Fig. 2b). This splice site mutation results in a premature stop codon 6 amino acids downstream (Fig. 2c), leading to protein truncation.

## Discussion

The study presented here describes the clinical and molecular genetic analysis of a Chinese family exhibiting Nance-Horan Syndrome (NHS). Mutations in the *NHS* gene have been reported from various populations throughout the world, including Turkish, Indian, Tunisian, Australian, Taiwanese, European and Chinese populations. Clinical evaluation of the available affected male subjects revealed typical NHS ocular and nonocular features that are more severe than in obligate carriers, in concordance with previous reports [8, 11, 17]. Both affected male subjects experienced profound vision loss, and one (II: 5) underwent cataract surgery. Mild mental retardation was exhibited by an affected male (III: 1) in this family. Mental retardation in NHS is highly variable and has been reported in 20–30% of affected subjects, which suggests it is a major component of the NHS syndrome [5].

NHS carrier females manifest mild and variable phenotypes. In one study, Y-sutural posterior lens opacities associated with microcornea and dental anomalies was observed in 100% of carrier females [18, 19], though carriers presenting clear to total lens opacities with small cornea, strabismus and nystagmus have also reported [11]. In our carrier females (II: 2; III: 7), cataract was no longer observed due to cataract surgery prior to the study. Although both carriers were of normal intellect, with normal facial features, microcornea, nystagmus, high myopia and strabismus were found.

Whole-exome sequencing followed by Sanger sequencing of variants identified a novel splice site mutation (c.1045 + 2T > A) in the *NHS* gene, mapping to the short arm of chromosome Xp22.13.

Splice site mutations represent a significant proportion of gene alterations leading to Mendelian disorders [20]. Such mutations influencing precursor mRNA splicing either result in complete skipping of one or more exons, retention of introns, creation of a pseudo-exon or activation of a cryptic splice site with in an exon or an intron [21]. Activation of cryptic splice site is the second most frequent consequence of splicing defect after exon skipping [22]. The identified sequence variant (c.1045 + 2T > A) in the *NHS* gene, located just after exon 4 at the 5′ consensus donor site, disrupts the natural splicing of exons 4-5.

The presence of aberrant splicing in the *NHS* gene caused by the NM_198270; c.1045 + 2T > A mutation was experimentally assessed by RT-PCR. Gel analysis showed and confirmed the presence of amplicons of two different lengths (421 bp, 837 bp) in a carrier female compared to a shorter amplicon (421 bp) in a control and a longer amplicon (837 bp) in an affected male. Sanger sequencing of cDNA confirmed the addition of 416 nucleotides to the mutated sequence.

The female fetus was at the 22^nd^ week of gestation at the time of sample collection for DNA extraction. Sequencing of the *NHS* gene revealed the splice site mutation in a heterozygous state (m/+). Sonographic scan of the fetus (data not shown) and follow-up ophthalmological examination after birth confirmed the presence of congenital cataract, in concordance with the molecular genetic analyses.

The NHS protein has multiple isoforms, and cell type-dependent differential expression is observed. The two major isoforms, NHS-A and NHS -1A, which are implicated in the pathogenesis of NHS, contain a functional WAVE homology domain at the N-terminus. This domain interacts with members of the Abelson-interactor (Abi) family and contributes to actin remodeling regulation and maintenance of cell morphology [9]. NHS-A, the neuronal and epithelial-specific isoform, is mainly expressed in the human eye lens, and the encoded protein associates with the peripheral cell membrane in the lens epithelium [23]. The identified splice site substitution in intron 4 induces a putative premature stop codon 7 codons downstream (p.G349-IVLVFSX), which is likely to result in a truncated NHS protein. Truncating mutations may lead to nonsense mediated decay (NMD), which results in complete loss of mutated proteins. However, it has been reported that some mRNAs harboring premature termination codons located in some specific regions can escape the NMD pathway, leading to an abnormal protein product [24]. Previously described RT-PCR analysis for a truncating mutation in exon 1(c.115C > T; Q39X) of the *NHS* gene in an Indian NHS family found no evidence of NMD pathway involvement [17]. In the present study, RT-PCR analysis depicting the presence of an aberrant mRNA transcript in the affected and carrier subjects also suggested escape from the NMD pathway. As a possible consequence, a truncated NHS protein composed of 355 amino acids with a damaging effect would be produced in the cells of these individuals, causing the disease phenotype. However, additional work is required to understand the exact pathogenic mechanism underlying NHS.

## Conclusions

In summary, whole-exome sequencing coupled with Sanger sequencing revealed a novel splice site mutation (NM_198270: c.1045 + 2T > A) in the 5’ consensus donor site of intron 4 in the *NHS* gene in a Chinese family. This mutation led to aberrantly spliced mRNA, which is likely to result in a truncated NHS protein. This work extends the genetic spectrum of NHS and contributes to knowledge of the molecular genetics involved. In addition, the present work signifies the importance of whole-exome analysis in gene discovery studies and the genetic-diagnosis of disease in the clinical examination as well.

## Additional files

Additional file 1: The overall statistics for variants of exome sequencing for the affected subject II:3. (DOCX 12 kb)
Additional file 2: Filtering statistics for the SNVs and InDels called from the exome sequencing data. (DOCX 12 kb)
Additional file 3: Candidate variations after filtering. (DOCX 12 kb)

## Abbreviations

EBV: Epstein-Barr virus
InDel: Insertions and deletions
IRB: Institutional Review Board
NHS: Nance-Horan syndrome
*NHS*: NHS gene
NMD: Nonsense mediated decay
SKLMG: State Key Laboratory of Medical Genetics
SNVs: Single nucleotide variants
WAVE: WASP family Verprolin-homologous
