# Supplementary material for: A novel NHS mutation causes Nance-Horan Syndrome in a Chinese family
Source: BMC Med Genet. 2017 Jan 7;18:2. doi: 10.1186/s12881-016-0360-9 (PMC5219716; doi:10.1186/s12881-016-0360-9)
Supplement: Additional file 1: — The overall statistics for variants of exome sequencing for the affected subject II:3. (DOCX 12 kb) [file 12881_2016_360_MOESM1_ESM.docx]

**The overall statistics for variants of exome sequencing for the affected subject II:3**

| Total | 37941628 (100%) |
| --- | --- |
| Properly mapped | 37678034 (99.31%) |
| Total_effective_reads | 37965040 |
| Total_effective_yield(Mb) | 4720.99 |
| Average_sequencing_depth_on_target | 56.10 |
| Mismatch_rate_in_all_effective_sequence | 0.26% |
| Coverage_of_target_region | 99.8% |
| Fraction_of_target_covered_with_at_least_20x | 89.4% |
| Fraction_of_target_covered_with_at_least_10x | 97.3% |
| Fraction_of_target_covered_with_at_least_4x | 99.4% |
