# Supplementary material for: A novel NHS mutation causes Nance-Horan Syndrome in a Chinese family
Source: BMC Med Genet. 2017 Jan 7;18:2. doi: 10.1186/s12881-016-0360-9 (PMC5219716; doi:10.1186/s12881-016-0360-9)
Supplement: Additional file 2: — Filtering statistics for the SNVs and InDels called from the exome sequencing data. (DOCX 12 kb) [file 12881_2016_360_MOESM2_ESM.docx]

**Filtering statistics for the SNVs and InDels called from the exome sequencing data**

| Sample ID | II:3 |
| --- | --- |
| Total coding InDels and SNVs | 24403 |
| NS, SS and InDels | 11992 |
| Low Frequency (MAF＜0.01) | 2226 |
| Rare variants in cataract-causing gene | 2 |

NS, non-synonymous variants; SS, splicing site variants; InDels, Insertion and Deletion variants
