# Supplementary material for: A novel NHS mutation causes Nance-Horan Syndrome in a Chinese family
Source: BMC Med Genet. 2017 Jan 7;18:2. doi: 10.1186/s12881-016-0360-9 (PMC5219716; doi:10.1186/s12881-016-0360-9)
Supplement: Additional file 3: — Candidate variations after filtering. (DOCX 12 kb) [file 12881_2016_360_MOESM3_ESM.docx]

**Candidate variations after filtering**

| Chr | Position | Ref_base | Alt_base | Hom/Het | Region | Gene | Annotation |
| --- | --- | --- | --- | --- | --- | --- | --- |
| Chr11 | 31822311 | C | T | Het | exonic | PAX6 | NM_000280: c.G451A:p.G151R |
| ChrX | 17739755 | T | A | Hom | splice site | NHS | NM_198270: exon4: c.1045+2T>A |
